# Supplementary material for: Leigh Syndrome in a Pedigree Harboring the m.1555A>G Mutation in the Mitochondrial 12S rRNA
Source: Genes (Basel). 2020 Aug 27;11(9):1007. doi: 10.3390/genes11091007 (PMC7565518; doi:10.3390/genes11091007)
Supplement: Supplementary file 1 [file genes-11-01007-s001.pdf]

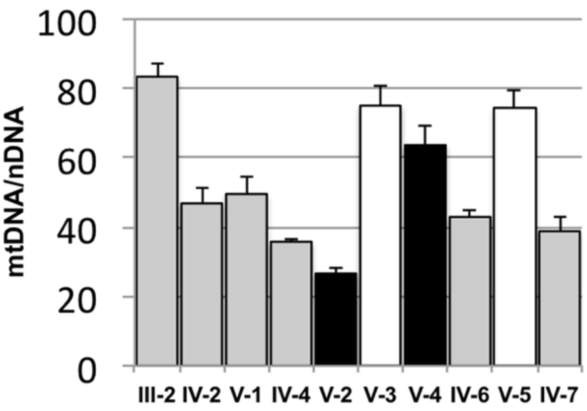

Figure S1. Blood mtDNA/nDNA ratio. White, grey and black colors code for healthy individuals, hearing loss and Leigh syndrome patients, respectively.

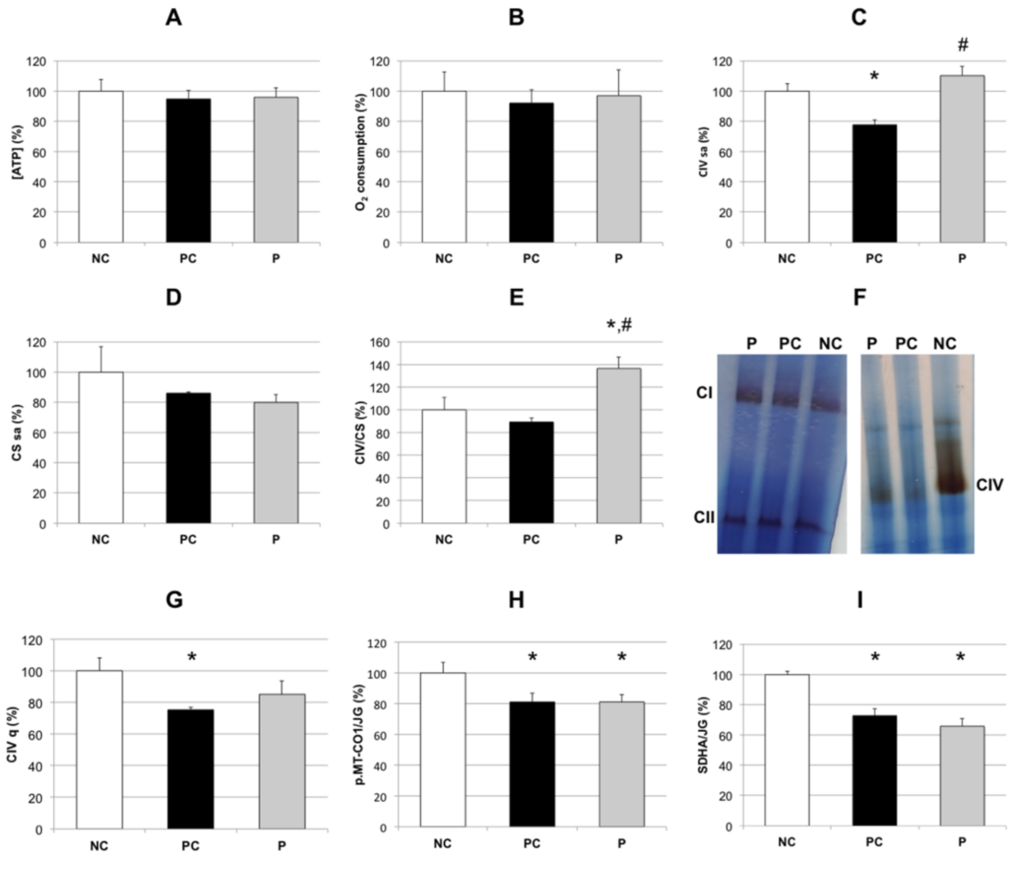

Figure S2. Cybrid analysis. NC (white), PC (grey) and P (black) code for negative control, positive control and patient. A) ATP concentration. B) Endogenous oxygen consumption. C) Respiratory complex IV (CIV) specific activity (sa). D) Citrate synthase (CS) sa. E) CIV/CS ratio. F) *In gel* complex I (CI), II (CII) and IV (CIV) activities. G) CIV quantity (q). H) p.MT-CO1/Janus Green (JG). I) SDHA/JG. *, # statistically significantly different from NC and PC, respectively.
